# Distribution Pattern of Suitable Areas and Corridor Identification of Endangered *Ephedra* Species in China

**DOI:** 10.3390/plants13060890

**Published:** 2024-03-20

**Authors:** Huayong Zhang, Jiangpeng Li, Hengchao Zou, Zhongyu Wang, Xinyu Zhu, Yihe Zhang, Zhao Liu

**Affiliations:** 1 Research Center for Engineering Ecology and Nonlinear Science, North China Electric Power University, Beijing 102206, China; 120212232021@ncepu.edu.cn (J.L.); zouhc@ncepu.edu.cn (H.Z.); zhy_wang@ncepu.edu.cn (Z.W.); 2 Theoretical Ecology and Engineering Ecology Research Group, School of Life Sciences, Shandong University, Qingdao 250100, China; liuzhao9555@sdu.edu.cn; 3 Dalian Eco-Environmental Affairs Service Center, No. 58 Lianshan Street, Shahekou District, Dalian 116026, China; suzaneilbeck@gmail.com; 4 School of Engineering, RMIT University, P.O. Box 71, Bundoora, VIC 3083, Australia; henin.zhang@rmit.edu.au

**Keywords:** distribution pattern, ecological corridors, *Ephedra*, MaxEnt model, MCR model

## Abstract

The suitable habitat of endangered *Ephedra* species has been severely threatened and affected by climate change and anthropogenic activities; however, their migration trends and restoration strategies are still relatively understudied. In this study, we utilized the MaxEnt model to simulate the suitable habitats of five endangered *Ephedra* species in China under current and future climate scenarios. Additionally, we identified significant ecological corridors by incorporating the minimum cumulative resistance (MCR) model. Under the current climate scenario, the suitable area of *Ephedra equisetina* Bunge, *Ephedra intermedia* Schrenk ex Mey, *Ephedra sinica* Stapf, and *Ephedra monosperma* Gmel ex Mey comprised 16% of the area in China, while *Ephedra rhytidosperma* Pachom comprised only 0.05%. The distribution patterns of these five *Ephedra* species were primarily influenced by altitude, salinity, temperature, and precipitation. Under future climate scenarios, the suitable areas of *E. equisetina*, *E. intermedia*, and *E. sinica* are projected to expand, while that of *E. monosperma* is expected to contract. Notably, *E. rhytidosperma* will lose its suitable area in the future. Our identified ecological corridors showed that the first-level corridors encompassed a wider geographical expanse, incorporating *E. equisetina*, *E. intermedia*, *E. sinica*, and *E. monosperma*, while that of *E. rhytidosperma* exhibited a shorter length and covered fewer geographical areas. Overall, our study provides novel insights into identifying priority protected areas and protection strategies targeting endangered *Ephedra* species.

## 1. Introduction

Environmental change profoundly impacts the structure and functioning of ecosystems across the biosphere, as well as the composition of biological communities and patterns of species distribution [1]. Climate change is pivotal in shaping plant species’ distribution patterns, which have been intricately linked to recurrent climatic fluctuations since the Fourth Great Ice Age [2,3]. Warming temperatures and altered rainfall regimes greatly affect species diversity and even increase the risk of plant extinction [4,5], posing significant challenges to the stability of China’s ecosystems and the conservation of endangered species [6,7]. Under the influence of global warming, plant distribution patterns are expected to undergo varying degrees of change, potentially leading to the extinction of 20–30% of species [8,9], particularly those that are endangered or have limited geographical ranges and are designated as national key protected plants [10]. Although higher temperatures may increase the genetic diversity of species and thus allow for the expansion of their ranges [11,12], species are losing their original habitats due to the overuse of resources by humans and changes in land use patterns [13]. Therefore, simulating the suitable distribution of species and identifying ecological corridors within the context of climate stress can serve as a theoretical foundation for formulating and implementing sustainable measures to conserve endangered species.

In recent years, the Species Distribution Model (SDM) has emerged as a pivotal approach used in hotspot studies for species distribution determination [14]. Among various species distribution models, the maximum entropy model (MaxEnt) is widely regarded as the most accurate and extensively employed model [15]. It offers several advantages, including its ability to yield reliable predictions with small sample sizes, flexible variable processing, and high precision in budget estimation [16]. With the advancement of MaxEnt, it has gained extensive application in safeguarding endangered flora, harnessing medicinal resources, and combating invasive species [17,18,19,20,21,22,23,24,25,26,27]. After predicting species-suitable areas, ecological corridors are good at connecting fragmented and isolated habitats [28,29]. The most commonly employed approach for studying migratory ecological corridors in species is integrating the minimum cumulative resistance (MCR) model with Geographic Information Systems (GISs) [30,31]. Notably, both the MaxEnt model and MCR model have been successfully utilized in conserving numerous endangered animal species [32,33], yielding significant outcomes. However, there remains an urgent need to investigate potential ecological corridors for plants.

*Ephedra* species are widely distributed in northern and northwestern China [13], with a total of 12 *Ephedra* species identified in the country [34]. Among them, *Ephedra equisetina* Bunge, *Ephedra intermedia* Schrenk ex Mey, *Ephedra sinica* Stapf, *Ephedra monosperma* Gmel ex Mey, and *Ephedra rhytidosperma* Pachom have been classified as endangered at varying levels of protection (see Section 4 for references). Notably, *E. rhytidosperma* is currently on the brink of extinction [35,36]. As a renowned sand fixation and traditional Chinese medicine species, it plays an irreplaceable role in maintaining ecological stability and treating diseases [37,38]. Therefore, it is imperative to comprehensively understand its distribution pattern of suitable areas and implement effective conservation measures for this genus. Despite extensive research on *Ephedra* species encompassing distribution surveys, physiological indicators, population structure, captive cultivation techniques, suitable area studies, and spatial distribution mapping [13,22,23,24,25,39,40,41,42,43,44], limited attention has been devoted to conservation design based on assessing potential habitat suitability. Henceforth, identifying appropriate suitable areas and dispersal ecological corridors for endangered *Ephedra* species under climate stress conditions are crucial to conserving and utilizing these valuable resources.

In this study, we employed the MaxEnt model to predict the suitable areas of five endangered *Ephedra* species (*E. equisetina*, *E. intermedia*, *E. sinica*, *E. monosperma*, and *E. rhytidosperma*). Utilizing these distribution areas as a basis, we utilized the MCR model to identify ecological corridors suitable for the dispersal of *Ephedra* species and optimize the habitat pattern. The specific purposes of this study are as follows: (1) determine the distribution of suitable areas for endangered *Ephedra* species under the current climatic conditions and the main environmental factors affecting their distribution; (2) predict and analyze the distribution of suitable areas and migration trends of endangered *Ephedra* species under changing climate scenarios in the future; and (3) calculate the minimum cumulative resistance of the ecological source area based on the MCR model to clarify the location of the ecological corridor. This study aims to provide a scientific foundation and practical guidance for the conservation of endangered *Ephedra* species and the preservation of biodiversity.

## 2. Results

### 2.1. Contemporary Distribution Patterns of Endangered Ephedra Species

**Figure 1:**
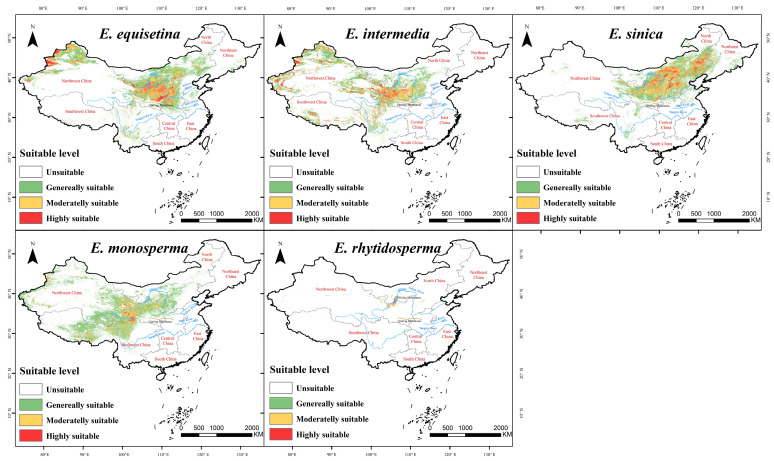
Spatial distribution of suitable areas of *E. equisetina*, *E. intermedia*, *E. sinica*, *E. monosperma*, and *E. rhytidosperma* under the current climate.

The MaxEnt model achieved AUC values of 0.901, 0.890, 0.903, 0.890, and 0.998 for the five endangered species of *Ephedra* in China (Figure S1). Over 16% of the total suitable areas are accounted for by the four species (*E. equisetina*, *E. intermedia*, *E. sinica*, and *E. monosperma*), with a certain degree of overlap and concentration being exhibited in their distributions, primarily in the southwestern part of North China, the eastern part of Northwest China, and the northern part of Central China (Figure 1). In contrast, *E. rhytidosperma* has an exceedingly small total suitable area, comprising merely 0.05%, predominantly concentrated within Ningxia’s Helan Mountain region (Figure 1 and Table 1). The key environmental factors influencing the spatial distribution of the suitable areas of *E. equisetina*, *E. intermedia*, *E. sinica*, *E. monosperma*, and *E. rhytidosperma* were topsoil calcium carbonate (16%), elevation (19.8%), precipitation of the wettest month (19.1%), elevation (33.1%), and topsoil pH (38.6%), respectively (Table 2).

### 2.2. Future Distribution Patterns of Endangered Ephedra Species

**Figure 2:**
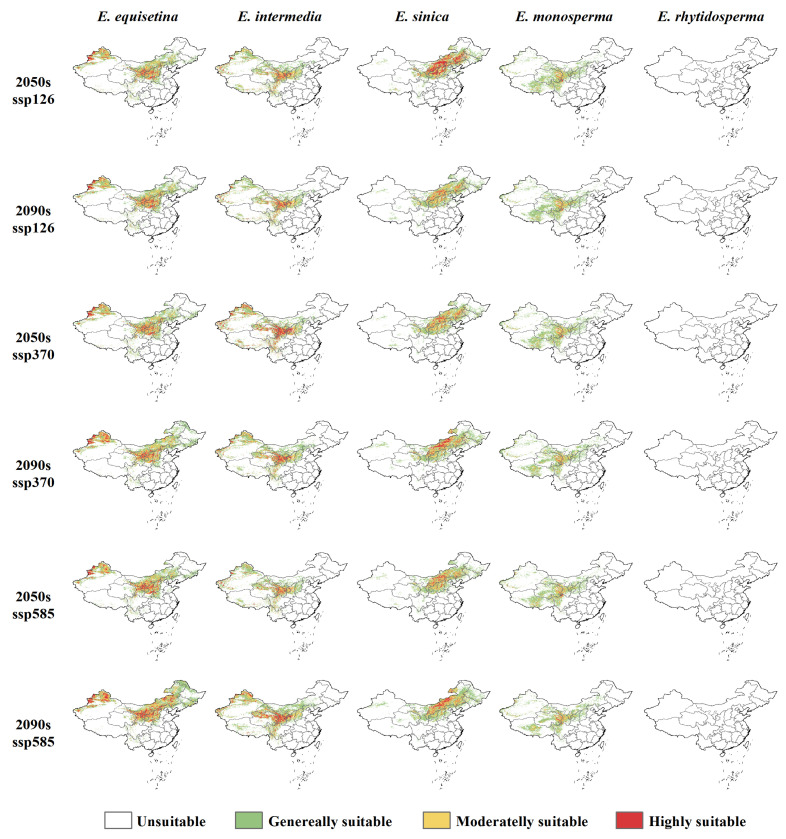
The spatial distribution of suitable areas of *E. equisetina*, *E. intermedia*, *E.sinica*, *E. monosperma*, and *E. rhytidosperma* under future climate scenarios.

Under the future (2050s and 2090s) climate scenarios (SSP126, SSP370, and SSP585), an expanding trend is shown in the suitable areas of *E. equisetina*, *E. intermedia*, and *E. sinica*, and a shrinking trend is shown in the areas of *E. monosperma*, while *E. rhytidosperma* completely loses its suitable areas (Figure 2 and Table S1). Among them, *E. equisetina* and *E. intermedia* have more suitable areas, mainly distributed in the areas north of the Qinling Mountains and northern Xinjiang. However, *E. sinica* mainly grows in North China and Northeast China. Thus, the highly suitable areas for *E. monosperma* are reduced to the Gansu and Qinghai provinces.

**Figure 3:**
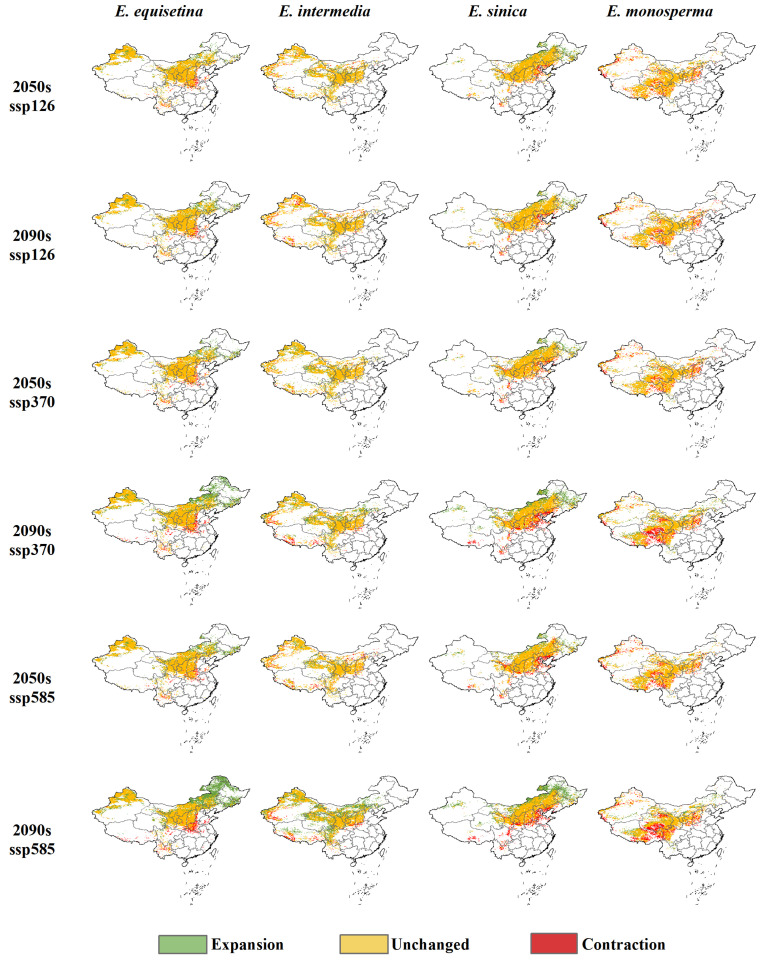
Distribution of suitable areas expansion and contraction of *E. equisetina*, *E. intermedia*, *E. sinica*, and *E. monosperma* under future climate scenarios in comparison to the current status.

The suitable areas of *E. equisetina* expand by more than 11% under future climate scenarios, reaching 51.12% in the SSP585 scenario (2090s) and expanding to Inner Mongolia and the three eastern provinces (Figure 3 and Table S1). *E. intermedia*’s suitable distribution area extends to limited areas in North China and northwest and southwest regions. According to the projections under the SSP585 scenario (2050s) and SSP126 scenario (2090s), the expansion of suitable areas for *E. intermedia* will not exceed 7%. However, alternative scenarios show its expansion surpassing 13%, with the most substantial increase observed in the SSP585 scenario (2090s), reaching up to 46.96%. The distribution of *E. sinica* will primarily expand northward. In the SSP585 scenario (2090s), its total suitable areas will reach its maximum value, whereas in the SSP126 scenario (2050s), the proportion of highly suitable areas is the highest, approaching 25%. The suitable areas for *E. monosperma* are projected to experience a reduction of over 5% under future climate scenarios, with a significant decrease of 12.42% anticipated under the SSP585 scenario (2050s). This decline is primarily observed across Qinghai, Tibet, Sichuan, Inner Mongolia, and Shanxi provinces.

### 2.3. Corridor Identification of Endangered Ephedra Species

**Figure 4:**
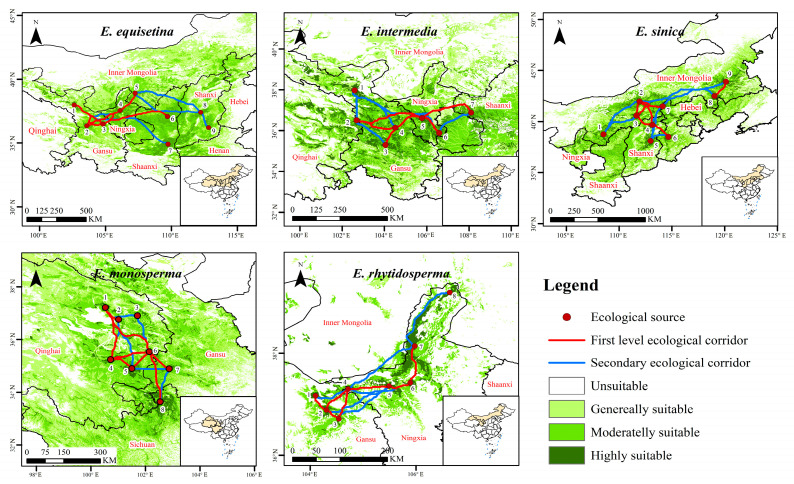
Ecological corridor identification of *E. equisetina*, *E. intermedia*, *E. sinica*, *E. monosperma*, and *E. rhytidosperma*.

The study area of *E. equisetina* in this research was situated in the eastern and northwestern regions of China. A total of nine ecological source sites were identified, along with the selection of 16 ecological corridors, including seven first-level corridors (Figure 4 and Table S2). These corridors primarily serve to connect various ecological source points in Northwest China. Notably, the longest ecological corridor spans from Jingyuan County in Gansu Province to Wenshui County in Shanxi Province, covering a remarkable distance of 625.475 km. Conversely, the shortest corridor stretches from Yongdeng County to Jingyuan County within Gansu Province, measuring a total length of 129.533 km. The research area of *E. intermedia* was primarily situated in the eastern part of the northwest region, encompassing a total of seven selected ecological source areas. Ultimately, thirteen optimal solutions were derived as diffusion ecological corridors, comprising six first-level corridors with a combined length of 1123.455 km and seven secondary corridors spanning a total distance of 1300.094 km. For *E. sinica*, nine ecological source areas were established within the North China research area. Among them, seven first-level diffusion corridors exclusively cover short distances, primarily situated within Qinghai province; additionally, eight secondary diffusion corridors exist, with the longest one extending from Uxin Banner in Inner Mongolia to Ar Horqin Banner in Inner Mongolia.

The study area of *E. monosperma* was primarily located in the Qinghai, Gansu, and Sichuan provinces. A total of eight ecological source sites were selected, resulting in the identification of 14 optimal solution diffusion ecological corridors. The cumulative length of these corridors amounted to 1959.849 km, with eight being classified as first-level corridors predominantly situated in Qinghai province. The study area of *E. rhytidosperma* was mainly concentrated in the northern part of Ningxia and the eastern part of Gansu province. Eight ecological source sites were established, leading to the identification of seven first-level corridors and six secondary corridors. These corridors had respective lengths totaling 389.031 km and 659.515 km, with their minimum cost distances primarily located in the southern part of Helan Mountain.

## 3. Discussion

In this study, all species within the endangered *Ephedra* genus in China exhibited AUC values ranging from 0.85 to 1, indicating a high level of reliability in our predictions and excellent performance of the model’s simulation [25,26].

The findings demonstrate that *E. equisetina*, *E. intermedia*, *E. sinica*, and *E. monosperma* have extensive distribution areas suitable for their growth under current climate models in China with a total proportion exceeding 16%, making them widely distributed species primarily concentrated in Gansu, Yunnan, Shaanxi, Shanxi, Hebei Henan, and northwestern Inner Mongolia. In contrast, the suitable area for *E. rhytidosperma* is relatively small at only 0.05%, mainly concentrated in the Helan Mountain region of Ningxia as a narrowly distributed species. These results are consistent with records in “Flora of China” and the conclusions drawn by most scholars regarding *Ephedra ‘s* distribution [25,35,36,38,39,40,41,42,43]. The highly suitable areas for these five endangered *Ephedra* species are primarily concentrated in Central–Western China while being relatively scarce or absent in southern regions due to differences in the climatic conditions between north and south [45].

In the arid regions of Northwest China, natural environmental factors such as salinity, temperature, and precipitation play pivotal roles in shaping the potential geographic distribution and growth development of species [17,20,44,46]. The findings from this study revealed that elevation, salinity, and temperature significantly influenced the distribution patterns of *E. equisetina*, *E. intermedia*, and *E. monosperma*. Additionally, precipitation, salinity, and elevation were pivotal in determining the distribution ranges of *E. sinica* and *E. rhytidosperma*. Among them, the primary environmental factor influencing *E. equisetina* was topsoil calcium carbonate, while for *E. rhytidosperma*, it was topsoil pH (H_2_O), both of which are closely associated with the physiological characteristics of medicinal and desert plants [25,39,47,48]. It has been demonstrated that osmoregulation plays a pivotal role in enhancing the water retention capacity under arid conditions [49]. Osmoregulation facilitates the accumulation of both organic and inorganic solutes, thereby reducing osmotic pressure and promoting plant metabolism [40]. Alkaloids, as crucial organic osmoregulators, are indispensable for effective osmoregulation [50]. The primary environmental determinant for *E. sinica* is the precipitation of the wettest month. Previous research has demonstrated that “extreme” precipitation significantly influences the growth of *E. sinica* [13,42], which aligns with the findings of this study. For *E. intermedia* and *E. monosperma*, elevation is the dominant environmental factor due to its association with significant variations in water availability, temperature, and light intensity. These changes at different altitudes create a more diverse habitat conducive to the survival of rare and endangered plant species [51].

Under future climate scenarios, the suitable areas for three *Ephedra* species (*E. equisetina*, *E. intermedia*, and *E. sinica*) are projected to increase significantly. Particularly under the SSP585 scenario in the 2090s, there is a substantial expansion of total suitable areas by 51.12%, 46.96%, and 19.61%, respectively, for these three species. This phenomenon can be attributed to warmer temperatures resulting in higher minimum temperatures and reduced frost damage, which lowers shrub mortality rates while creating favorable conditions for their expansion and colonization [52,53,54,55]. Moderate temperature warming, along with increased precipitation, positively influence the dispersal and population growth of these species [55,56]. Studies indicate that surface temperatures in China will rise by 2.7–2.9 °C under the future global warming context, accompanied by an average annual increase in precipitation by 20%, particularly in the northern and northwestern regions [57,58]. Chinese scholars have also found that increased rainfall combined with rising temperatures will have a positive impact on the suitable habitats of these three *Ephedra* species, corroborating our study’s findings [25,39,40,41,42].

Climate change will result in the expansion of the suitable distribution areas for certain wide-ranging and invasive species while simultaneously reducing the size of suitable distribution areas for narrow-ranging and rare species [59]. Drastic climate change may even lead to the fragmentation of species distributions and habitat loss, thereby posing a greater threat to endangered species with limited natural ranges and exacerbating their risk of extinction [60]. Under projected future climate scenarios, *E. monosperma* is expected to undergo habitat reduction while *E. rhytidosperma* faces a complete loss of its habitat, highlighting the urgent need for conservation research. The decrease in suitable areas for *E. monosperma* under different climate scenarios can be attributed to the multifaceted impact of temperature, which exerts a more pronounced negative influence on vegetation than precipitation, particularly as temperatures continue to rise [60,61,62,63,64]. Previous studies have identified a negative correlation between rare and endangered plant species, such as *Sinowilsonia henryi* Hemsl, *E. rhytidosperma*, and *Phoebe bournei* Yen C Yang, with annual precipitation and average temperature. Moreover, future habitat predictions indicate that *E. rhytidosperma* is projected to experience a complete loss of its suitable distribution area, which corroborates the findings of this study [65]. Meanwhile, *E. rhytidosperma* exhibits a restricted distribution, confined solely to the flood fan area or shallow mountain foothills of Helan Mountain, characterized by a limited range and small population size in an unfavorable growth environment [35,36,65]. With recent land development and utilization activities in the eastern foothills of Helan Mountain, the habitat of *E. rhytidosperma* has suffered severe degradation, exacerbating its endangered status.

Previous research has demonstrated that, in response to future warming temperatures, most species are expected to undergo latitudinal shifts toward higher latitudes [19,20,66]. However, due to variations in their suitable capacities toward environmental changes, different species exhibit distinct migration patterns [67]. In the SSP370 scenario, suitable areas for *E. equisetina*, *E. intermedia*, *E. sinica*, and *E. monosperma* are projected to shift toward higher latitudes. Similarly, under the SSP585 scenario, suitable areas will also move poleward (with *E. intermedia* and *E. monosperma* shifting equatorward by the 2050s and 2090s, respectively). Conversely, only *E. equisetina* and *E. sinica* migrate to higher latitudes in the SSP126 scenario (2050s). These findings highlight that there are differences in direction and distance with respect to suitable areas among various species within the genus *Ephedra* under climate stress conditions. This confirms that the migratory abilities of species constitute one factor influencing their adaptability to future changes [66].

Ecological corridors play a crucial role in addressing wildlife and plant habitat fragmentation issues by breaking down biological isolation barriers, facilitating gene exchange among populations, mitigating biodiversity impacts caused by fragmented habitats, and further improving ecosystem services [13,64,68,69,70,71]. Our research findings indicate that habitats connected by ecological corridors are more effective in preserving local species than isolated patches [13,64,69,71]. Due to the dispersed growth characteristics of species in the *Ephedra* genus, establishing individual protected areas becomes challenging [72]. Therefore, the identification and establishment of ecological corridors can provide a more effective means of protecting *Ephedra* species. These ecological corridors not only consider the distance between points but also account for cumulative environmental resistance effects on *Ephedra* plant dispersal, enhancing their operational feasibility [73]. Moreover, this positive impact becomes stronger over time and plays a crucial role in the maintenance of local biodiversity, as well as the promotion of ecosystem balance and stability [74].

Notably, the selected five endangered *Ephedra* species exhibited largely unaffected ecological corridors by environmental factors such as topography and slope. At the same time, they are also situated at a considerable distance from residential areas [13]. Since *Ephedra* is a plant with a relatively weak dispersal ability, we prioritized short-range corridors in the selection process as important ecological corridors. However, this study predicted suitable areas for *Ephedra* under future climate stress conditions, and as a result, long-distance corridors were also chosen [13,72].

Based on the outcomes of assessing the adaptability of endangered *Ephedra* species to climate-change-induced stress, suitable areas for *E. equisetina* in Henan and Hebei will shrink in future climate scenarios but expand toward Inner Mongolia and the three eastern provinces. Consequently, over time, the service value of three ecological corridors extending from Etoke Banner in Inner Mongolia to Zichang City in Shaanxi Province, from Etoke Banner in Inner Mongolia to Wenshui County in Shanxi Province, and from Jingyuan County in Gansu Province to Wenshui County in Shanxi Province will progressively gain significance. Therefore, it is crucial to prioritize their construction and restoration [25]. In the future climate scenario, more suitable areas for *E. intermedia* will be concentrated in Gansu, Ningxia, and Qinghai but will also expand into Inner Mongolia and Shaanxi. Henceforth, it is imperative to enhance the restoration of ecological corridors in Inner Mongolia within the existing corridor design [24]. Under future climate change scenarios, the suitable areas and high-suitableness areas of *E. sinica* will shift northward, resulting in a decrease in suitable areas within Shanxi and Hebei provinces. Consequently, the value of ecological corridors in Shanxi and Hebei provinces will decrease in value under future climate conditions. Therefore, it is crucial to prioritize constructing ecological corridors within Inner Mongolia, specifically from Siziwang Banner to Ar Horqin Banner, from Wengniute Banner to Ar Horqin Banner, and from Shangyi County in Hebei Province to Wengniute Banner in Inner Mongolia [13,39]. Regarding *E. monosperma* under future climate conditions, its highly suitable areas are still concentrated at the junction of the Qinghai, Gansu, and Sichuan provinces. Corridor construction should be dominated by the first-level corridor and supplemented by the secondary corridor [24,64,68,75,76]. *E. rhytidosperma* has a small distribution area and low population size and is highly endangered. A single corridor restoration can no longer satisfy the protection and management needs of the *Ephedra* species. On this basis, as suggested for other threatened rare plant species with a very restricted range or very small populations [77], protection is fundamental. In our case, this can be carried out by taking measures such as establishing nature reserves for special protection and management, maintaining the integrity of the habitat, and avoiding further fragmentation of the habitat caused by human factors [35,36].

## 4. Materials and Methods

### 4.1. Data Sources and Data Processing

According to the “National Key Protected Wild Plants” (2021 edition) and the “China Rare and Endangered Plant Information System-Protection of Wild Plants in Provinces and Municipalities”, there are five rare and endangered species of Ephedra in China [78,79]: *E. equisetina*, *E. intermedia*, *E. sinica*, *E. monosperma*, and *E. rhytidosperma*. Distribution data for these species were obtained from the Chinese Virtual Herbarium (CVH: https://www.cvh.ac.cn (accessed on 6 April 2023)), the Global Biodiversity Information Facility (GBIF: https://www.gbif.org (accessed on 6 April 2023)), and the Species Diversity Data Platform (http://www.especies.cn (accessed on 6 April 2023)). To prevent overfitting caused by dense sample distribution, ENMTools (http://purl.oclc.org/enmtools (accessed on 7 April 2023)) were used to eliminate duplicate distribution points while retaining only one valid point within a 1 km grid [80,81].

The base map of China used in this study was acquired from the National Center for Basic Geographic Information (https://ngcc.cn/ngcc (accessed on 6 April 2023)). The contemporary climate data were obtained from WorldClim (https://worldclim.org (accessed on 6 April 2023)) with a spatial resolution of 1 km, encompassing 19 bioclimatic variables. The elevation factors were obtained from WorldClim (https://worldclim.org (accessed on 6 April 2023)) with a spatial resolution of 1 km, and the slope and aspect data were extracted by using the surface analysis tool of ArcGIS 10.8.2. The soil factor data were obtained from the World Soil Database (http://www.iiasa.ac.at/web/home/research/researchPrograms/water/HWSD.html (accessed on 6 April 2023)) constructed by the Food and Agriculture Organization of the United Nations and the International Institute for Applied Systems Analysis, Vienna, with a spatial resolution of 1 km. The annual China Land Cover Dataset (CLCD) derived from Landsat and produced by Yang et al. is based on the GEE platform [82], with a spatial resolution of 30 m. NDVI data were obtained from the Research Center for Resource and Environmental Sciences of the Chinese Academy of Sciences (https://www.resdc.cn (accessed on 6 April 2023)) with a resolution of 1 km and annual averages from 2010 to 2020.

The future climate data (19 bioclimatic variables) were obtained from WorldClim version 2.1 (https://worldclim.org (accessed on 6 April 2023)) with a spatial resolution of 1 km, including the base period (1970–2000) and future (2050s: 2041–2060 and 2090s: 2081–2100) climate data. Three future climate scenarios (SSP126, SSP370, and SSP585) representing low, medium, and high CO_2_ emission scenarios were selected. Due to the lack of data on future topography and soils under climate change, this study defaults to no change in the nation’s topography and soils over the projected time period [22].

The environmental variables utilized in the MaxEnt and MCR models were adjusted to a spatial resolution of 1 km by using ArcGIS 10.8.2. Three categories of environmental factors, namely climate, elevation, and soil (Table 3), were selected for inclusion in the MaxEnt (version 3.4.4) model. In order to mitigate the adverse impact of multicollinearity among similar environmental factors on model overfitting, which can compromise the accuracy and precision of the results, this study extracted 36 environmental factors and conducted a Pearson correlation analysis by using IBM SPSS 27 software. If a strong correlation (R^2^ > 0.8) was identified between similar factors, the factor with the lowest percentage contribution was eliminated [22,25,27]. The MCR model incorporates four environmental variables, namely elevation, slope, land use type, and NDVI, to determine the minimum resistance surface influencing the dispersal of the *Ephedra* species [13].

### 4.2. Suitable Habitat Evaluation

The screened environmental factors and the natural distribution point data of five rare and endangered *Ephedra* species were imported into the MaxEnt model, and a value of 75% of the distribution point data was randomly selected as a training set for building the model, with the remaining 25% used as a test set for verification [83]. The results were output after being repeated 10 times, the output format was Logistic, and the rest of the options were adopted in the model default settings. The accuracy of the model prediction results was examined by using the AUC, which is 0–1 [84]. It is generally believed that AUC < 0.6 indicates that the prediction results fail, 0.6 ≤ AUC ≤ 0.8 indicates that the prediction results are generally accurate, 0.8 < AUC ≤ 0.9 indicates that the prediction results are better, and AUC > 0.9 indicates that the prediction results are excellent [85,86].

Using tools for reclassification in ArcGIS, the maximum test sensitivity plus specificity threshold (MTSPS) was used as a boundary to divide the suitable habitat into four levels: unsuitable areas, *p* ≤ MTSPS; generally suitable areas, MTSPS < *p* ≤ 0.5; moderately suitable areas, 0.5 < *p* ≤ 0.75; and highly suitable areas, *p* > 0.75 [87]. The ArcGIS10.8.2 tool was used to simulate and analyze the changes in the suitable areas of five endangered *Ephedra* species in different periods. The expansion area, contraction area, and retention area can be obtained by overlapping the current and future grid maps to analyze the change in the suitable areas over time.

### 4.3. MCR Modeling for Corridor Identification

Utilizing the MCR model, this study identifies ecological corridors that are conducive to the dispersal of five endangered *Ephedra* species. The model elucidates the movement process of these species from their source to target locations by identifying the most efficient or least resource-intensive path required to overcome resistance. A lower level of resistance facilitates smoother ecological flow [68,70,76].

This study combines the data of highly suitable and moderately suitable areas for *E. equisetina*, *E. intermedia*, *E. sinica*, *E. monosperma*, and *E. rhytidosperma* generated by the MaxEnt model as the fundamental data source for running the MCR model [13,88]. The relatively independent regions in these base data are characterized and classified, and attributes are extracted to form individual ecological source sites. Subsequently, the rasterized evaluation results are converted into vector polygons and transformed into individual ecological source points by using feature-to-point tools. By utilizing the cost distance module in ArcGIS 10.8.2, the cost distances and cost backlinkages between each pair of ecological source points are calculated to simulate the minimum cumulative resistance paths formed among them as a basis for identifying ecological corridors [89]. Next, four environmental variables including elevation, slope, land use type, and NDVI were employed to determine the minimum resistance surface affecting *Ephedra* species dispersal. The resistance gradient of the *Ephedra* species was divided into six resistance classes (1–6), with the highest resistance class being 6 and the smallest resistance class being 1 [72]. A correlation matrix of the resistance values was established by analyzing the resistance values between factors by using the statistical analysis software yaahp (version 10.3) [75,90]. Finally, weights for each resistance factor were calculated [32].

The gravity model can be employed to quantitatively assess the magnitude of interaction between sources and targets, thereby identifying crucial corridors [91]. In this study, we employed the gravity model to calculate the interaction forces among various ecological source points and delineated the resistance value range by using a natural breakpoint method [92]. The significance of ecological corridors for five Ephedra species was classified into first-level and secondary corridors and general ecological corridors. General ecological corridors were not considered in this study due to their long distances and high resistance values [13,23,24].

## 5. Conclusions

In this study, we employed the MaxEnt model to predict the distribution patterns of five endangered *Ephedra* species (*E. equisetina*, *E. intermedia*, *E. sinica*, *E. monosperma*, and *E. rhytidosperma*) under current and future climate scenarios. Additionally, using the MCR model, we identified the minimum resistance corridors between ecological source sites while quantitatively analyzing the corridor classes by applying the gravity model. The following conclusions can be drawn:

(1) Under the current climate scenario, four species, namely *E. equisetina*, *E. intermedia*, *E. sinica*, and *E. monosperma*, inhabit over 16% of their suitable area in China. Conversely, *E. rhytidosperma* occupies a comparatively smaller proportion of its suitable areas in China at only 0.05%. Altitude was the most critical factor limiting the growth of *E. intermedia* and *E. monosperma*, while salinity played a pivotal role in constraining the growth of *E. equisetina* and *E. rhytidosperma*. In contrast, the distribution of *E. sinica* exhibited a stronger dependence on precipitation factors.
(2) Under the future climate scenario, the expansion of the suitable area of *E. equisetina*, *E. intermedia*, and *E. sinica* was maximized under the SSP585 scenario (2090s). The suitable area of *E. monosperma* shrank, with the greatest degree of shrinkage in the SSP585 scenario (2050s), by 12.42%. In contrast, *E. rhytidosperma* loses its suitable area under future climate scenarios.
(3) By employing the MCR model and gravity model, we successfully identified 71 crucial ecological corridors for five Ephedra species, all of which are strategically located away from anthropogenic surfaces. These corridors play a pivotal role in ensuring the long-term survival of *Ephedra* species. Identifying these ecological corridors provides valuable insights into the conservation and management strategies for *Ephedra* species.

In conclusion, *E. equisetina*, *E. intermedia*, and *E. sinica* demonstrate promising potential for development under future climate scenarios, while *E. monosperma* and *E. rhytidosperma* face unfavorable prospects or even the risk of extinction. This study provides valuable scientific guidance for the conservation planning of these five *Ephedra* species by understanding their habitats and identifying potential ecological corridors to promote sustainable development and biodiversity conservation.

## Figures and Tables

**Table 1:** Suitable areas of *E. equisetina*, *E. intermedia*, *E. sinica*, *E. monosperma*, and *E. rhytidosperma* under the current climate.

Species	Generally Suitable Areas /×10^4^ km^2^	Moderately Suitable Areas /×10^4^ km^2^	Highly Suitable Areas /×10^4^ km^2^	Total Suitable Areas	
				/×10^4^ km^2^	Percentage/%
*E* *. equisetina*	107.90	47.29	16.40	171.60	17.9
*E* *. intermedia*	134.55	62.24	25.30	222.10	16.1
*E. sinica*	89.77	50.84	14.11	154.72	16.1
*E* *. monosperma*	124.43	34.16	30.51	161.64	16.8
*E* *. rhytidosperma*	1.79	2.05	0.64	4.48	0.05

**Table 2:** Percent contribution of main environmental factors.

Species	Driving Factor	Contribution
*E* *. equisetina*	T_CACO_3_: topsoil calcium carbonate	16
	Elevation	14.5
	Bio6: min temperature of coldest month	11.7
	Bio2: mean diurnal range	10.4
	Bio12: annual precipitation	10
*E* *. intermedia*	Elevation	19.8
	T_BS: topsoil base saturation	14.2
	Bio15: precipitation seasonality	10.6
	Bio9: mean temperature of driest quarter	9.5
	T_CACO_3_: topsoil calcium carbonate	7.8
*E* *. sinica*	Bio13: precipitation of wettest month	19.1
	T_CACO_3_: topsoil calcium carbonate	15.8
	Bio19: precipitation of coldest quarter	13
	Elevation	10.9
	Bio15: precipitation seasonality	10.9
*E* *. monosperma*	Elevation	33.1
	Bio2: mean diurnal range	12.4
	Slope	8.9
	Bio19: precipitation of coldest quarter	8.8
	Bio12: annual precipitation	6.7
*E. rhytidosperma*	T_PH_H_2_O: topsoil pH (H_2_O)	38.6
	Bio12: annual precipitation	15.5
	Bio11: mean temperature of coldest quarter	13.3
	Aspect	10.2
	T_CASO_4_: topsoil gypsum	7.9

**Table 3:** Environment variables driving MaxEnt.

Symbol	Environmental Factors	Unit	Symbol	Environmental Factors	Unit
Bio1	Annual mean temperature	°C	Bio19	Precipitation of coldest quarter	mm
Bio2	Mean diurnal range	°C	Elev	Elevation	m
Bio3	Isothermality	\	Aspect	Aspect	°
Bio4	Temperature seasonality	\	Slope	Slope	°
Bio5	Max temperature of warmest month	°C	T_PH_H_2_O	Topsoil pH (H_2_O)	−log (H^+^)
Bio6	Min temperature of coldest month	°C	T_GRAVEL	Topsoil gravel content	%
Bio7	Temperature annual range	°C	T_SILT	Topsoil silt fraction	%
Bio8	Mean temperature of wettest quarter	°C	T_CLAY	Topsoil clay fraction	%
Bio9	Mean temperature of driest quarter	°C	T_SAND	Topsoil sand fraction	%
Bio10	Mean temperature of warmest quarter	°C	T_OC	Topsoil organic carbon	%
Bio11	Mean temperature of coldest quarter	°C	T_CEC_CLAY	Topsoil CEC (clay)	cmol/kg
Bio12	Annual precipitation	mm	T_CEC_SOIL	Topsoil CEC (soil)	cmol/kg
Bio13	Precipitation of wettest month	mm	T_BS	Topsoil base saturation	%
Bio14	Precipitation of driest month	mm	T_TEB	Topsoil TEB	cmol/kg
Bio15	Precipitation seasonality	\	T_CACO_3_	Topsoil calcium carbonate	%
Bio16	Precipitation of wettest quarter	mm	T_CASO_4_	Topsoil gypsum	%
Bio17	Precipitation of driest quarter	mm	T_ESP	Topsoil sodicity (ESP)	%
Bio18	Precipitation of warmest quarter	mm	T_ECE	Topsoil salinity (Elco)	dS/m

## Data Availability

All links to the input data are reported in the manuscript, and all the output data are available upon request to the authors.

## Supplementary Materials

The following supporting information can be downloaded at https://www.mdpi.com/article/10.3390/plants13060890/s1: Figure S1. AUC training values for *E. equisetina* (a), *E. intermedia* (b), *E. sinica* (c), *E. monosperma* (d), and *E. rhytidosperma* (f). Table S1. Spatial distribution of suitable areas changes of *E. equisetina*, *E. intermedia*, *E. sinica*, *E. monosperma*, and *E. rhytidosperma* under future climate scenarios. Table S2. Ecological source coordinates of endangered *Ephedra* species.
