# Supplementary material for: Distribution Pattern of Suitable Areas and Corridor Identification of Endangered Ephedra Species in China
Source: Plants (Basel). 2024 Mar 20;13(6):890. doi: 10.3390/plants13060890 (PMC10975542; doi:10.3390/plants13060890)
Supplement: Supplementary file 1 [file plants-13-00890-s001.zip › plants-2890067-supplementary.pdf]

plants

MDPI

Supplementary Materials

Distribution Pattern of Suitable Areas and Corridor Identification of Endangered *Ephedra* Species in China

Huayong Zhang [1,2,*], Jiangpeng Li [1], Hengchao Zou [1], Zhongyu Wang [1], Xinyu Zhu [3], Yihe Zhang [4] and Zhao Liu [2]

1. Research Center for Engineering Ecology and Nonlinear Science, North China Electric Power University, Beijing 102206, China; 120212232021@ncepu.edu.cn (J.L.); zouhc@ncepu.edu.cn (H.Z.); zhy_wang@ncepu.edu.cn (Z.W.)
2. Theoretical Ecology and Engineering Ecology Research Group, School of Life Sciences, Shandong University, Qingdao 250100, China; liuzhao9555@sdu.edu.cn
3. Dalian Eco-Environmental Affairs Service Center, 116026, No. 58 Lianshan Street, Shahekou District, Dalian 116026, China; suzaneilbeck@gmail.com
4. School of Engineering, RMIT University, P.O. Box 71, Bundoora, VIC 3083, Australia; hen-in.zhang@rmit.edu.au

* Correspondence: zhanghuayong@sdu.edu.cn; Tel.: +86-010-61773936

This word file includes

Figure and Table Legends

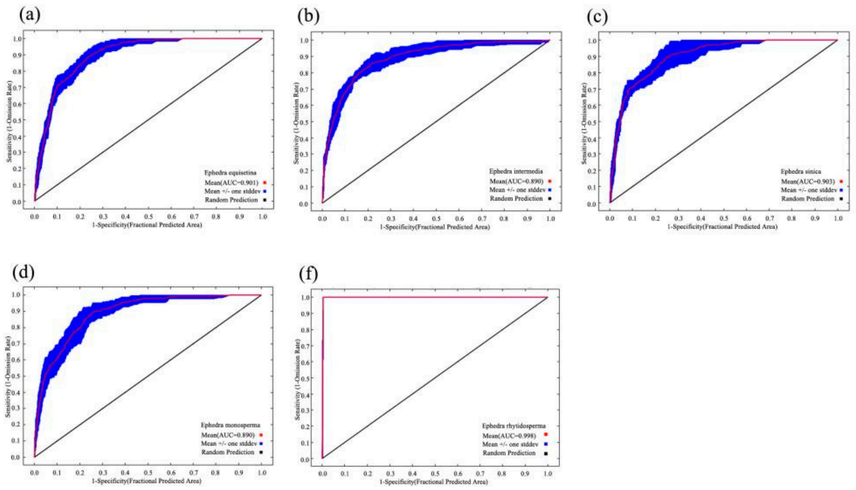

Figure S1. AUC training values for *E. equisetina* (**a**), *E. intermedia* (**b**), E. sinica (**c**), *E. monosperma* (**d**), and *E. rhytidosperma* (**f**).

Table S1. Spatial distribution of suitable areas changes of *E. equisetina*, *E. intermedia*, *E. sinica*, *E. monosperma*, and *E. rhytidosperma* under future climate scenarios.

Species	Scenarios	Generally Suitable Area/×10⁴km²/ Increase or Decrease/ ×10⁴ km²/ Change/%	Moderately Suitable Area/ ×10⁴ km²/ Increase or Decrease/ ×10⁴ km²/ Change/%	Highly Suitable Area/ ×10⁴ km²/ Increase or Decrease/ ×10⁴ km²/ Change/%	Total Suitable Area/ ×10⁴ km²/ Increase or Decrease/ ×10⁴ km²/ Change/%
E. equisetina	Current climate	107.90	47.29	16.40	171.59
	2050s, SSP126	106.59/−1.31/ −1.21	60.88/13.59/ 28.74	24.19/7.79/ 47.5	191.66/20.07/ 11.70
	2050s, SSP370	110.48/2.58/ 2.39	60.33/13.04/ 27.57	24.82/8.42/ 51.34	195.63/24.04/ 14.01
	2050s, SSP585	110.62/2.72/ 2.52	65.35/18.06/ 38.19	27.01/10.7/ 65.24	202.98/31.39/ 18.29

	2090s, SSP126	110.07/2.17/2.01	61.51/14.22/30.07	24.39/7.99/48.72	195.97/24.38/14.21
	2090s, SSP370	123.92/16.02/14.85	75.33/28.04/59.29	29.36/12.96/79.02	228.61/57.02/33.23
	2090s, SSP585	127.93/20.03/18.56	85.86/38.57/81.56	45.52/29.12/177.56	259.31/87.72/51.12
E. intermedia	Current climate	93.70	43.34	17.62	154.66
	2050s, SSP126	110.38/16.68/17.80	48.70/5.36/12.37	17.16/−0.46/−2.61	176.24/21.58/13.95
	2050s, SSP370	98.22/4.52/4.82	46.95/3.61/8.33	42.65/25.03/142.05	187.82/33.16/21.44
	2050s, SSP585	103.88/10.18/10.86	43.39/0.05/0.11	17.53/−0.09/−0.51	164.81/10.15/6.56
	2090s, SSP126	99.58/5.88/6.28	43.83/0.49/1.13	18.22/0.60/3.41	161.63/6.97/4.51
	2090s, SSP370	114.15/20.45/21.82	52.10/8.76/20.21	22.66/5.04/28.60	188.91/34.25/22.15
	2090s, SSP585	128.61/34.91/37.26	66.08/22.74/52.47	32.59/14.97/84.96	227.29/72.63/46.96
E. sinica	Current climate	89.77	50.84	14.11	154.72
	2050s, SSP126	85.67/−4.10/−4.57	47.02/−3.82/−7.51	42.80/28.69/203.33	175.49/20.77/13.42
	2050s, SSP370	98.78/9.01/10.03	51.69/0.85/1.67	13.53/−0.58/−4.11	164.01/9.29/6.00
	2050s, SSP585	97.61/7.84/8.73	46.82/−4.02/−7.91	13.14/−0.97/−6.87	157.58/2.86/1.84
	2090s, SSP126	99.41/9.64/10.74	50.25/−0.59/−1.16	11.73/−2.38/−16.87	161.39/6.67/4.31
	2090s, SSP370	106.04/16.27/18.12	50.14/−0.70/−1.38	19.62/5.51/39.05	175.81/21.09/13.63
	2090s, SSP585	112.40/22.63/25.21	54.44/−0.40/−0.79	18.22/4.11/29.13	185.06/30.34/19.61
E.monosperma	Current climate	124.43	34.16	3.05	161.64
	2050s, SSP126	110.77/−13.6/−10.98	30.05/−4.11/−12.03	3.57/0.52/17.05	144.39/−17.25/−10.67
	2050s, SSP370	112.71/−11.7/−9.42	31.66/−2.50/−7.32	3.48/0.43/14.10	147.84/−13.80/−8.54
	2050s, SSP585	105.33/−19.1/−15.35	31.75/−2.41/−7.06	4.49/1.44/47.21	141.57/−20.07 −12.42
	2090s, SSP126	113.76/−10.6/−8.58	28.96/−5.20/−15.22	3.29/0.24/7.87	146.01/−15.63/−9.67
	2090s, SSP370	115.75/−8.68/−6.97	31.29/−2.87/−8.40	4.10/1.05/32.43	151.14/−10.50/−6.50
	2090s, SSP585	107.83/−16.6/−13.34	32.25/−1.91/−5.59	4.34/1.29/42.30	144.42/−17.22/−10.65
E.rhytidosperma	Current climate	1.79	2.05	0.64	4.48
	2050s, SSP126	0.00/−1.79/−100.00	0.00/−2.05/−100.00	0.00/−0.64/−100.00	0.00/−4.48/−100.00
	2050s, SSP370	0.00/−1.79/−100.00	0.00/−2.05/−100.00	0.00/−0.64/−100.00	0.00/−4.48/−100.00
	2050s, SSP585	0.00/−1.79/−100.00	0.00/−2.05/−100.00	0.00/−0.64/−100.00	0.00/−4.48/−100.00
	2090s, SSP126	0.00/−1.79/−100.00	0.00/−2.05/−100.00	0.00/−0.64/−100.00	0.00/−4.48/−100.00
	2090s, SSP370	0.00/−1.79/−100.00	0.00/−2.05/−100.00	0.00/−0.64/−100.00	0.00/−4.48/−100.00
	2090s, SSP585	0.00/−1.79/−100.00	0.00/−2.05/−100.00	0.00/−0.64/−100.00	0.00/−4.48/−100.00

Table S2. Ecological source coordinates of endangered *Ephedra* species.

Species	Coordinates of Ecological Sources	Location

E. equisetina (9)	37.97N，106.31E	Liangzhou district, Gansu Province
	36.36N，106.15E	Yongdeng County, Gansu Province
	36.51N，107.13E	Jingyuan County, Gansu Province
	37.54N，106.12E	Hongsi Pu District, Ningxia
	38.91N，107.25E	Etoke Banner, Inner Mongolia
	37.08N，109.68E	Zichang City, Shaanxi Province
	34.89N，109.74E	Pucheng County, Shaanxi Province
	37.40N，112.23E	Wenshui County, Shanxi Province
	36.24N，112.82E	Zhangzi County, Shanxi Province
E. intermedia (7)	37.97N，102.58E	Liangzhou district, Gansu Province
	36.50N，102.71E	Ledu District, Qinghai Province
	35.31N，104.08E	Lintao County, Gansu Province
	36.14N，104.50E	Yuzhong County, Gansu Province
	36.63N，105.71E	Haiyuan County, Ningxia
	35.87N，106.60E	Pengyang County, Ningxia
	36.87N，108.09E	Wuqi County, Shaanxi Province
E. sinica (9)	38.76N，108.52E	Uxin Banner, Inner Mongolia
	41.98N，111.90E	Siziwang Banner, Inner Mongolia
	40.59N，111.65E	Tumd East Banner, Inner Mongolia
	39.68N，113.14E	Ying County, Shanxi Province
	38.12N，112.97E	Meng County, Shanxi Province
	38.52N，114.67E	Quyang County, Hebei Province
	41.50N，114.13E	Shangyi County, Hebei Province
	42.51N，119.03E	Wengniute Banner, Inner Mongolia
	43.92N，120.12E	Ar Horqin Banner, Inner Mongolia
E. monosperma (8)	37.24N，100.54E	Haiyan County, Qinghai Province
	36.77N，101.03E	Hangyuan County, Qinghai Province
	36.90N，101.71E	Datong Hui and Tu Autonomous County, Qinghai Province
	35.24N，100.72E	Tongde County, Qinghai Province
	34.90N，101.50E	Zeku County, Qinghai Province
	35.53N，100.72E	Tongren City, Qinghai Province
	34.88N，102.87E	Xiahe County, Gansu Province
	33.64N，102.55E	Ruoergi County, Sichuan Province
E. rhytidosperma (8)	37.17N，104.10E	Jingtai County, Gansu Province
	36.89N，104.31E	Jingtai County, Gansu Province
	36.72N，104.54E	Jingyuan County, Gansu Province
	37.30N，104.72E	Shapotou District, Ningxia
	37.37N，105.49E	Shapotou District, Ningxia
	37.43N，105.90E	Hongsi Pu District, Ningxia
	38.14N，105.98E	Qingtongxia City, Ningxia
	39.20N，106.64E	Huinong District, Ningxia
